# Tumor Banks: A Quality Control Scheme Proposal

**DOI:** 10.3389/fmed.2019.00225

**Published:** 2019-10-17

**Authors:** Ligia Craciun, Selim Alex Spinette, Marc Rassy, Roberto Salgado, Alexandre de Wind, Pieter Demetter, Laurine Verset, Maria Gomez-Galdon, Marie Chintinne, Nicolas Sirtaine, Nicolas de St Aubain, Ioanna Laios, Francoise Roy, Denis Larsimont

**Affiliations:** ^1^Tumor Bank, Institut Jules Bordet, Brussels, Belgium; ^2^Department of Anatomical Pathology, Jules Bordet Institute, Brussels, Belgium

**Keywords:** biobank, electrophoretic integrity, quality control, DNA, RNA, morphology, quality scores

## Abstract

**Introduction:** Tumor banks make a considerable contribution to translational research. Using emerging molecular tests on frozen material facilitates the development of new diagnostic and therapeutic strategies, especially in rare cases. However, standard quality control schemes are lacking in the current literature.

**Methods:** In 2017, we have conducted a robust quality control test on 100 of 15,000 fresh frozen samples collected between 2000 and 2013 at the Jules Bordet Tumor Bank (Brussels). RNA and DNA extraction was done. The quality of RNA, DNA and proteins were evaluated, respectively by measuring RNA Integrity Number (RIN), by checking Electrophoretic Integrity (EI) and by performing Immunohistochemistry staining (IHC). A score, ranging from poor (1) to excellent (4), was attributed based on technical analysis.

**Results:** RNA purity was scored 4 in 97% of the cases, 3 in 2%, and 2 in 1%. RIN scores were similarly 4 in 89%, 3 in 10%, and 2 in 1% of the cases. DNA purity was scored 4 in 94% and 3 in 6%, EI was scored 4 in 100% of the cases. Despite morphology loss after freezing, HER2, ER, and Ki67 IHC stainings yielded a score of 4 in the majority of samples. Furthermore, participating in the ISBER Proficiency Testing helped us validate our techniques and the technician's work. Seven processing schemes were carried out, the scores obtained were very satisfactory (20/27) or satisfactory (7/27).

**Conclusion:** Tumor Banks can be precious for translational research. Nevertheless, firm quality controls should be applied to ensure high quality material delivery. Only then can biobanks contribute to diagnostics, biomarkers discovery and reliable molecular test development.

## Introduction

Collecting samples for research is an old concept among pathologists and researchers. Nevertheless, biobanking is only fairly recent (1), as well as legislation concerning collection of human tissue and data protection (EU Data Protection Directive—Directive 95/46/EC). Controlling the quality of collected material in a biobank is crucial before providing tissue specimens for research. Quality control procedures must be in place to evaluate the samples and the effects of long-term storage. The lack of reproducibility in gene signatures is often associated with tissue heterogeneity due to, among other things, the lack of standardization of collection procedures (2). A good level of molecular integrity is essential to avoid variability in the results of research projects. The quality of nucleic acids is of major importance for several techniques used in genetic analysis.

Convenient quality control procedures must check the validity of final products (samples or derivatives) for different applications of end-use in research, irrespective of the used extraction method. Scores and cutoffs are to be adopted to determine the quality acceptance limits.

The first phase, termed pre-analytical phase, summarizes all steps from tissue sampling to the start of the desired end-use application. Each of these steps can affect the quality of the sample, the quality of the results, and their reproducibility (3, 4). Once the critical pre-analytical steps (medications, anesthesia, warm and cold ischemia time) for an application are known, researchers will only examine samples that meet the pre-analytical conditions previously defined. Quality control procedures (QC) are used to either confirm tissue quality from known pre-analytical conditions or investigate tissue quality from unknown pre-analytical condition(s). The ideal quality control “biomarkers” should be ubiquitous, measurable by accessible methods and leading to a dichotomous response to a specific pre-analytic variation.

Validation of clinically appropriate biomarkers should take into account the potential impact of pre-analytical variation on each of them. This validation process is the key to research using bio-resources (a requalified tissue sample for research and its associated data). Rapid stabilization of tissues by snap freezing immediately can reduce artifactually altered gene expression. Moreover, unlike FFPE tissue, the RNA and DNA from frozen tissue are of high molecular weight, lack cross-linking modifications and are therefore better candidates for the “next-generation” testing.

Good QC tools aim to test the molecular integrity and protein quality. They must also be compatible with genomic, epigenomic, transcriptomic, proteomic, and metabolomic tests.

Moreover, histological control of stored tissue is a crucial step. Generally, 10% of the frozen samples are unsuitable for the molecular analysis mainly because of insufficient quantity of malignant cells or necrosis (5).

The purpose of this work is to establish the quality limits of the tissues stored in tumor banks by independently evaluating the morphological (proteins) and molecular (DNA and RNA) characteristics on randomized selected frozen tumor samples.

In parallel, we have used the Biospecimen Proficiency Testing (PT) programme launched by IBBL, as an external quality assessment tool to verify the precision and accuracy of the in house biospecimens testing methods. Seven processing and testing schemes were performed: DNA Extraction from FFPE Material, DNA Extraction from Frozen Tissue, DNA Quantification and Purity, Total RNA Extraction from Frozen Tissue, RNA Integrity, RNA Quantification and Purity, and Tissue Histology.

## Materials and Methods

### Ethical Approval

Ethical approval, concerning the biobank activities and objectives, was granted by the ethics committee of the Institut Jules Bordet (CE1891 and CE2897). Of note, and according to the Belgian laws (2008-12-19/44 and 2018-01-09/14), the ethics committee of the Institut Jules Bordet approved the study protocol and waived the requirement of patients written consent.

### Biospecimens

One hundred biobank frozen samples (52 breasts, 13 thyroids, 12 lymph nodes, 9 endometrium, 3 ovaries, 2 sarcomas, 2 kidney, 2 colon, 1 prostate, 1 lung, 1 small intestine, 1 spleen, 1 uterus) originating from 100 patients were tested for DNA, RNA and protein quality. Selected samples dated from 2000 to 2013. Tumor samples were embedded in Tissue-Tek® O.C.T.™ Compound (by Sakura®) and frozen at −80°C. This method allows sectioning on a cryostat without residues during the staining procedure. Indeed, frozen sections were performed on a cryostat (by Leica Biosystems®). The first slide was stained by H&E (Hematoxylin and Eosin). Twenty serial sections were collected in RNase free Eppendorf tubes. Four additional sections were used for the IHC staining. Of note, all necessary material for sections handling was cooled on dry ice, to preserve the cold chain and avoid temperature fluctuations. The tumor morphology and cellularity were last reviewed by a pathologist.

### DNA/RNA Extraction

DNA and RNA were extracted from frozen specimens using AllPrep DNA/RNA Micro Kit (Qiagen) according to the manufacturer's instructions. DNA and RNA were finally eluted in a volume of 20 μL.

#### DNA Quality Assessment

A good DNA quality is important for studies on genomic DNA and CGH analyses. Two parameters were evaluated: concentration and purity, measured by the OD and DNA integrity by electrophoresis gel. The ratio for pure DNA should be between 1.8 and 2.1: a lower ratio is indicative of protein contamination, while a higher ratio indicates a degradation of the DNA. This ratio is only an indication of purity of nucleic acids and does not necessarily reflect the integrity of the nucleic acids.

### DNA Gel Analysis

A visual analysis on electrophoresis gel was done to estimate the sample integrity. The degree of DNA degradation was examined using electrophoresis in a 2% agarose gel (Reliant™ Gel System, 2% SeaKem® Gold Agarose, Lonza, USA). Intact genomic DNA appears as a compact, high-molecular-weight band with no scanty low-molecular-weight smears. 1 kb DNA ladder from Solis Biodyne was used as molecular marker. A quality score could be assigned (Table 1).

**Table 1:** Electrophoresis integrity (EI) quality scores.

**Quality**	**Ratio 260/280**	**Electrophoresis integrity (EI)**	**Score**
Bad	1.2–1.4	Smear of 2 kb	1
Poor	1.4–1.6	Smear of 5 kb	2
Good	1.6–1.8	Smear of 10 kb	3
Very good	1.8–2.1	Single band of high molecular weight	4

#### RNA Quality Assessment

The yield and purity of total DNA and RNA were determined using a spectrophotometer (Nanodrop TM ND-1000, Thermo Fisher Scientific). A 260/280 OD ratio >1.8 was considered an indicator of acceptably pure RNA, relatively free of protein.

### RNA Integrity Number

RNA was examined on the Agilent 2100 bioanalyzer, based on microfluidic capillary electrophoresis. RNA 6000 Nano LabChip kits were used. For each sample, 1 μL of extracted RNA was analyzed. RIN scores, ranging from 1 to 10, were retrieved. A RIN between 7 and 10 was associated with intact RNA.

### RNA Purity and Integrity Score

Based on the 260/280 OD ratio and on the RIN, a score was assigned for each case, as shown in Table 2.

**Table 2:** RNA purity and integrity score attribution based on 260/280 OD Ratio and RIN.

**Quality**	**260/280 OD Ratio**	**RIN**	**Score**
Bad	1.2–1.4	1–4	1
Poor	1.4–1.6	1–4	2
Good	1.6–1.8	4.1–6.9	3
Very good	1.8–2.1	7.0–10.0	4

### Immunohistochemistry Staining

Consecutive frozen tissue sections (4 μm) were immunohistochemically (IHC)-stained using a BenchMark XT IHC/ISH automated slide stainer (Ventana Mediated Systems, by Roche®). The following antibodies were used: anti-HER2/NEU (rabbit monoclonal antibody, clone 4B5, Roche® Ventana®); anti-Estrogen Receptor (ER) (rabbit monoclonal antibody, clone SP1, Roche® Ventana®); and anti-Ki-67 (mouse monoclonal antibody, clone MIB-1, Agilent®). Breast tumor samples (*n* = 52) were tested with anti-HER2/NEU and anti-ER antibodies. Non-breast tumor samples (*n* = 48) were tested with anti-Ki67 antibody.

### Morphological and Proteins Quality

For this study, H&E staining allowed the evaluation of cellular integrity and morphology, while immunohistochemistry staining (Ki67, HER2, and ER) provided a practical evaluation of proteins quality control.

A quality score, based on visual evaluation of quality staining, was assigned. All scoring systems were based on two separate components: the specificity and the intensity of staining. Technical sensitivity and specificity cannot be accurately calculated when IHC is used as a qualitative test because it is merely a descriptive test. The relation between the staining and the protein availability isn't linear. Calibration controls aren't either available. Scoring was blindly done by two independent pathologists. While scoring, routine sections from FFPE (formalin fixed and paraffin embedded) blocks were used as reference (Table 3).

**Table 3:** Visual evaluation of specificity and intensity of the IHC staining.

**Quality**	**Visual evaluation**	**Score**
Bad	Low specificity/Low intensity	1
Poor	Low specificity/Moderate intensity	2
Good	Moderate specificity/Moderate intensity	3
Very good	high specificity/High intensity	4

### ISBER Proficiency Testing

#### DNA Extraction From FFPE Cells Scheme

The material used for this scheme was a Jurkat cell line. We received one tube containing 2 FFPE sections of 20 μm. We extracted the DNA following our usual routine silica membrane-based DNA extraction method. The extracted DNA sample was shipped back to the PT provider. The total DNA yield per 20 μm section, DNA purity, DNA integrity (DIN), DNA functionality and amplifiability (cross-linking assessment) and DNA quality (ENZO score) of all extracted DNA were assessed.

#### DNA Extraction From Frozen Tissue Scheme

The material used for this scheme was a pig (*Sus*) liver. We received one tube containing one CryoXtract core of 10 to 20 mg. We performed the DNA extraction following our usual routine silica membrane-based DNA extraction method. The extracted DNA sample was shipped back to the PT provider. The total DNA yield per mg of tissue, the DNA purity (A260/A280), the double-stranded DNA yield per mg of tissue, the DNA integrity (DIN) and the presence of PCR inhibitors using a SPUD assay were assessed by IBBL.

#### DNA Quantification and Purity Scheme

The DNA used for this scheme was extracted from whole blood. We received three different Test Items containing DNA at a different concentration and 260/280 ratio (i.e., Tube A, Tube B, and Tube C). For each Test Item (Tube A, Tube B, and Tube C), we measured the DNA concentration (μg/ml) and 260/280 ratio by spectrophotometry.

#### RNA Extraction From Frozen Tissue Scheme

The material used for this scheme was a pig (*Sus*) liver. In this scheme, we received one single “Processing Item” (one tube containing one CryoXtract core of 10 to 20 mg). The RNA was extracted following our usual routine silica membrane-based RNA extraction method. The extracted RNA sample was shipped back to the PT provider. The total RNA yield per mg of tissue, the RNA purity (A260/A280) and the RNA integrity (RIN) were assessed by IBBL.

#### RNA Integrity Scheme

The RNA used for this scheme was extracted from a Jurkat cell line by a silica-based method. Three different Test Items containing RNA at a different level of integrity (i.e., Tube A, Tube B, and Tube C) were received. For each Test Item (Tube A, Tube B, and Tube C), we measured the RNA Integrity (RIN) on the Agilent® 2100 Bioanalyzer System.

#### RNA Quantification and Purity Scheme

The RNA used for this scheme was extracted from a Jurkat cell line by a silica-based method. Three different Test Items containing RNA at a different concentration and 260/280 ratio (i.e., Tube A, Tube B, and Tube C) were received. For each Test Item (Tube A, Tube B, and Tube C), we measured the RNA concentration (μg/ml) and 260/280 ratio by spectrophotometry.

#### Tissue Histology Scheme

The Test Items were pictures of human colon adenocarcinoma (Test Items A) and human breast adenocarcinoma (Test Item B, Test Item C, Test Item D, and Test Item E). The tissue characterization/mapping was done through the assessment of the percentage of uninvolved tissue areas (Test Item A, Test Item B, and Test Item C) and of viable tumor areas (Test Item D and Test Item E).

For each test, the scoring system was based on deviation from the assigned value. A consensus score was established as follow: below 1 standard deviation: 0 (very satisfactory); below 2 standard deviations: 1 (satisfactory); above 2 standard deviations: 2 (questionable); and above 3 standard deviations: 3 (requiring action). The results were reported through the website http://biospecimenpt.ibbl.lu.

## Results

### Total DNA Quality Control

The morphology was successfully determined in the majority of samples. Two samples were tumor free and one has been totally consumed through sectioning.

**Figure 1:**
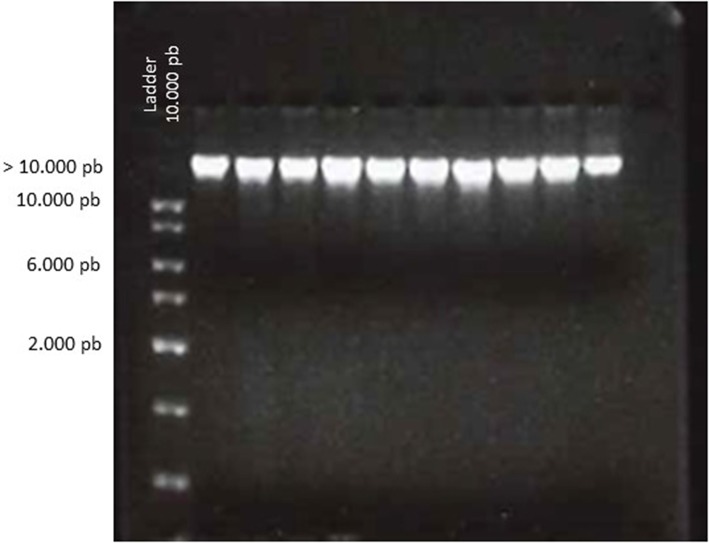
Electrophoretic analysis of genomic DNA from biobanked frozen tumor samples. DNA (5 μL) was loaded on a 2% agarose gel and visualized by ethidium bromide staining. The gel shows the result of 10 representative samples. Compact bands of DNA were observed for all samples at a high molecular weight according to the ladder. The absence of smearing favors the absence of DNA degradation.

Based on 260/280 ratio, the majority of tested samples were evaluated with a score of 4 (94%) (Figure 1), and 6 samples were scored with 3. The 260/230 ratio were used as a secondary measure of nucleic acid purity. The data is available but doesn't provide any supplementary information. No contamination by salt or organic compounds was noted. The EI was estimated at score 4 for all the tested samples.

### Total RNA Quality Control

**Figure 2:**
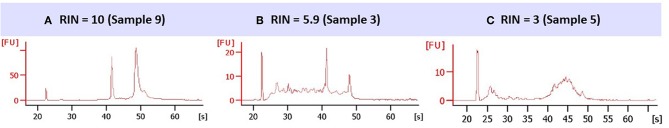
Representative electropherogram for different RIN classes. 1 μL of sample RNA was charged in the microfabricated chips. **(A)** RIN = 10, from one representative sample classified as score 4; the different regions (pre-, 5S-, fast-, inter-, precursor-, post-region) and peaks (marker, 18S, 28S) are correctly presented. **(B)** RIN = 5.9, from one representative sample classified as score 3; intermediate peaks appear on the zone 5S and fast-regions, pointing to RNA degradation. **(C)** RIN = 3, from one representative sample classified as score 2; peaks of ribosomal subunits, 18S and 28S, are absent.

Upon optical density (OD) measurement of extracted RNA, most samples were evaluated with a score of 4 (97%), two samples were scored with 3 and one sample was unusable due to insufficient RNA amount. In the latter, the tissue fragment was mainly fibrotic on microscopic examination. RIN values (Figure 2) were classified as of sufficient quality: score of 4 (89%) and score of 3 (10%); the sample characterized by a score of 2 (1%) was considered inadequate for further analysis.

### Assessment of Morphological and Proteins Quality

**Figure 3:**
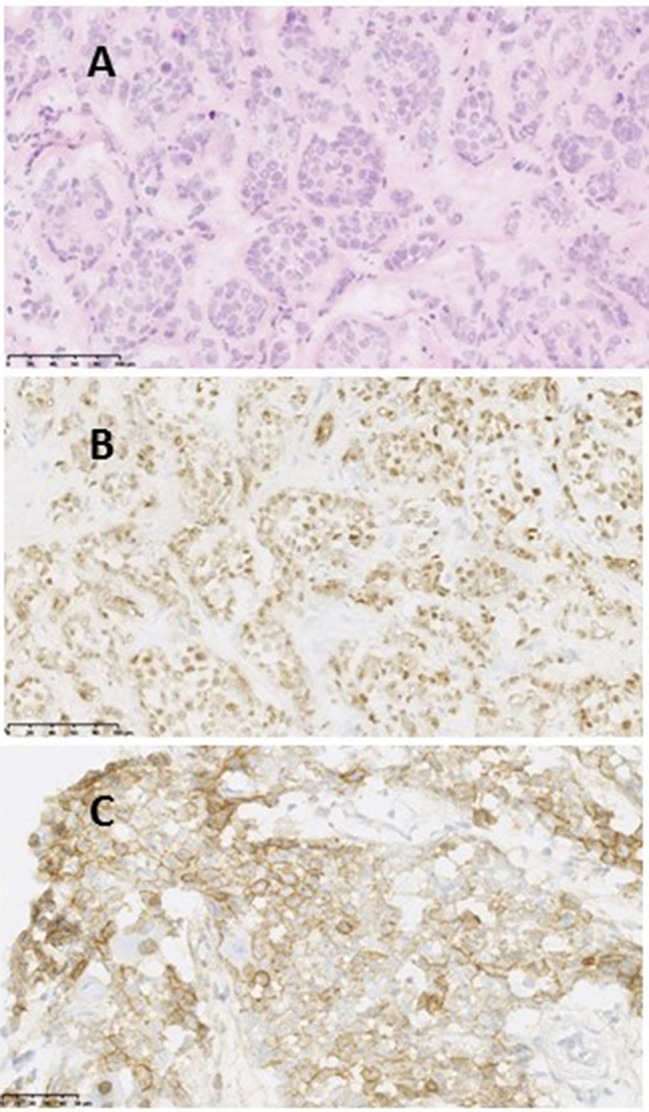
Frozen breast tumor sample. **(A)** Hematoxylin/eosin staining (30x); **(B)** ER IHC staining; **(C)** Her2 IHC staining. The tumor was characterized as ductal carcinoma, ER/Her2 positive, by the pathologist in 2001 and confirmed on the 16-year-old frozen sample. The pathologist evaluation is based on four criteria: the intensity of staining, the percentage of positive cells, background, and the localization of hybridized antibody (membrane, cytoplasm).

All tested samples were characterized by a good histologic quality control. The percentage of area of the tissue involved with tumor was considered acceptable despite the presence of freezing artifacts in almost all cases (Figure 3). The majority of screened samples were scored with 3 or 4 (Table 4).

**Table 4:** Summary of the IHC scores assigned to samples of the study cohort based on the visual evaluation.

**IHC staining**	**Score 4 (%)**	**Score 3 (%)**	**Score 2 (%)**	**Score 1 (%)**
Her2	64	31	5	0
ER	57	33	10	0
Ki67	82	16	0	2

### Proficiency Testing Report

#### DNA Extraction From FFPE Cells

Our results (16,990 ng/20 μm slice) were compared to all the results' average (7,453.22 ng/20 μm slice) and silica membrane-based (8,141.92 ng/ 20 μm slice). They have been designated as “accurate” or “very satisfactory,” consensus score “0.” The ratio 260/280 (1.95) has been designated as “accurate” or “very satisfactory,” consensus score “0.” The DIN (5.40) was also designated as “accurate” or “very satisfactory,” score 0, compared to all results average (4.49). The ENZO score was qualified as good-excellent and the level of PCR inhibitors were qualified as compatible with CGH assay.

#### DNA Extraction From Frozen Tissue Scheme

Our results (1,733.60 ng/mg tissue) were compared to all results average (1,865.18 ng/mg tissue) and silica membrane-based (2,045.98 ng/mg tissue). They have been designated as “accurate” or “very satisfactory,” consensus score “0.” The double-stranded DNA yield per mg tissue were 1,134.60 ng/mg tissue. It was considered “very satisfactory” when compared with the mean of all results (955.83 ng/mg tissue). The ratio 260/280 (1.90) has been designated as “accurate” or “very satisfactory,” consensus score “0.” The DIN (6.30) was also designated as “accurate” or “very satisfactory,” score 0, compared to all results average (5.67).

#### DNA Quantification and Purity

The accuracy of our measurements was qualified as “very satisfactory,” consensus score “0” when compared with mean values: Tubes A/B/C, 246.1/117.6/ 31.5 vs. 248.5/119.5/32.7 μg/ml. DNA purity was evaluated as “very satisfactory,” consensus score “0”: obtained values were 1.71/1.30/1.83 compared with expected ratio values: 1.72/1.32/1.92.

#### RNA Extraction From Frozen Tissue Scheme

The average of all expected results was 1,843.68 ng/mg tissue, our result was 572.9 ng/mg tissue, designated “acceptable” or “satisfactory,” consensus score “1.” RNA purity was evaluated as “very satisfactory,” consensus score “0”: obtained ratio value was 2.1 compared with expected ratio value 2.03. The obtained RIN value was 6.8 instead 6.55 mean all values considered “very satisfactory,” consensus score “0.”

#### RNA Integrity Scheme

The three obtained values (2.73/4.33/9.47) were slightly different from expected values (2.55/4.74/9.43), yielding a consensus score of “0”/“1”/“0.”

#### RNA Quantification and Purity Scheme

The three obtained values (93.6/60.1/33.4 μg/ml) were slightly different from expected values (90.5/60.1/33.4), yielding a consensus score of “0”/“0”/“1.” RNA purity was evaluated as “satisfactory” and “very satisfactory,” data not shown.

#### Tissue Histology Scheme

Regarding tissue histology, our consensus score required revision for the slide A, colon adenocarcinoma, and was satisfactory or very satisfactory for slides B and C, breast carcinoma. Evaluating viable tumor tissue was satisfactory or very satisfactory for slides D and E.

## Discussion

The Institut Jules Bordet tumor bank is completely integrated in the Pathological Department. The pathologist and technician pathologist are critical to identify the presence and type of tumor lesion and are responsible for ensuring diagnostic use prior to releasing tissue for research. The proximity of the operating room allows specimens quick handling reducing pre-analytical biases. Some samples stored in our biobank are already more than 20 years old.

Medical research projects are dependent on biobanked tissue of high quality because the gene expression analysis is affected by the quality of extracted RNA and DNA (6). Different factors influence the quality of nucleic acids and proteins including pre-analytical variables, transport, duration of processing at ambient temperature, necrosis, temperature and freezing products, size and number of aliquots and storage duration. The long-term storage temperature could impact the tissue quality. We currently use the OCT embedding medium on cryovials, followed by −80°C storage temperature for the solid tumors. The OCT embedding medium acts as cryoprotector from the freeze-thaw effects and gives the possibility to verify the histology after frozen sectioning and H&E staining.

Histologic quality control must be performed on biological samples. Different percentage cut-offs of tumor nuclei are required for each downstream use of the sample. The percentage of viable tumor cells is very important to perform NGS. It can range from 2% (7) to 80% (8). In our opinion, it is crucial to inform the researcher on the histologic quality before performing sensitive and expensive techniques.

RNA preservation is particularly important for gene expression analysis. RNA is known to be quickly degraded by ubiquitous RNase enzymes. OD reading is useful for determining the amount and purity of nucleic acids. Ribosomal RNA integrity is often used to reflect all RNAs physical integrity. In our study, RIN values were evaluated at score 4, even for the oldest samples. In addition to rRNA which represent 80% of total RNA, the messenger RNA (mRNA) and microRNA (miRNA) which constitute a small class of coding and, respectively, noncoding cellular RNA are the most interesting target for research. The stability of mRNA is better despite complete degradation of rRNA (9). Once again, the researcher together with biobank staff has to establish the tissue quality requirements before starting the research project. If the rare tumors are concerned by the research, too stringent criteria must be revised.

We have determined the suggested applications based on the value of the RIN (Table 5) (10).

**Table 5:** Suggested molecular biology applications based on the value of the RIN.

**RIN**	**Suggested application**
1–4	PCR Amplification of small fragments
4.1–6.9	qRT-PCR applications
7.0–10.0	Any application evaluating gene expression

At our institute, optical density and gel electrophoresis are commonly adopted for quick evaluation of extracted DNA purity and integrity. On a 2% agarose gel, intact genomic DNA appears as a compact, high-molecular-weight band with no low-molecular-weight smears. The scores assigned to our 2017 QC were mostly of 4, demonstrating an excellent quality of stored frozen tissues. Amplifying a specific sequence by PCR could give an information about usability of DNA for downstream molecular applications. Low amounts of PCR products can be attributed to poor quality DNA or poor quality tissues.

Investigation of different surface proteins can yield useful information on pathological pathways or biomarkers related to a particular disease. Specific immunohistochemical stains can be performed to evaluate specific antigens. This technique is routinely used on FFPE diagnostic blocs.

Assessing protein integrity is important since the freezing process may result in proteolysis and protein degradation (11, 12). Nevertheless, an accessible comprehensive way to assess protein quality is not available for frozen samples. Histologic evaluation of the tissue by the pathologist can provide a preliminary screening of degraded tissues. Our method fits with our lab equipment. Evaluating proteins quality by IHC of frozen tissue is really challenging because of cell structure freezing-related modifications. Training or experience is required for the pathologist scoring the stained slides. Mass spectrometry has become a crucial technique for almost all proteomics experiments, it should be considered for further analysis. It represents, indeed, the gold standard technique to test the protein quality, this technique is judicious when available. Using one or other technique is depending on the laboratory equipment and possibilities.

External quality tests, such as the ISBER Biorepository proficiency testing (13), allow both validation and improvement of protocols. Every failed QC item is deeply analyzed and corrected. If necessary, a dialogue with the external partner is established for additional information. Data can be exchanged regarding the test performance or the technician's work. The protocol deviation is then registered and corrective action adopted.

## Conclusion

In conclusion, we proposed in this paper an easy quality control schema of biobank stored frozen samples with different ages, different tissue types and different types of morphology. Quality control for RNA, DNA and proteins might be performed periodically on a subset of samples in a biobank. The quality of our tumor samples was very satisfactory and adapted to a large panel of “next-generation” technologies. Our methods and techniques were validated by the external ISBER Proficiency testing program. Based on easy scoring procedures, the biobanks can give indications for downstream molecular biology application (14).

## Data Availability Statement

The datasets generated for this study are available on request to the corresponding author.

## Author Contributions

LC conceived and designed the experiment. SS performed the techniques. LC, SS, MR, RS, AW, PD, LV, MG-G, MC, NS, NSA, and DL analyzed the data. IL and FR quality management supervisor. LC and MR wrote the paper. DL oversaw the project.

### Conflict of Interest

The authors declare that the research was conducted in the absence of any commercial or financial relationships that could be construed as a potential conflict of interest.
